# Recurrent *Clostridioides difficile* Infection and Outcome of Fecal Microbiota Transplantation Use: A Population-Based Assessment

**DOI:** 10.1093/ofid/ofae309

**Published:** 2024-06-15

**Authors:** Nirja Mehta, Dana Goodenough, Nitin K Gupta, Stepy Thomas, Christina Mehta, Radhika Prakash, Michael H Woodworth, Colleen S Kraft, Scott K Fridkin

**Affiliations:** Department of Medicine, Division of Infectious Diseases, Emory University School of Medicine, Atlanta, Georgia, USA; Georgia Emerging Infections Program, Decatur, Georgia, USA; Georgia Emerging Infections Program, Decatur, Georgia, USA; Atlanta Veterans’ Affairs Medical Center, Decatur, Georgia, USA; Emory University School of Medicine, Atlanta, Georgia, USA; Atlanta Gastroenterology Associates, Georgia, USA; United Digestive, Atlanta, Georgia, USA; Northside Hospital, Department of Gastroenterology, Atlanta, Georgia, USA; Georgia Emerging Infections Program, Decatur, Georgia, USA; Atlanta Veterans’ Affairs Medical Center, Decatur, Georgia, USA; Emory University School of Medicine, Atlanta, Georgia, USA; Department of Medicine, Division of Infectious Diseases, Emory University School of Medicine, Atlanta, Georgia, USA; Department of Medicine, Division of Infectious Diseases, Emory University School of Medicine, Atlanta, Georgia, USA; Department of Medicine, Division of Infectious Diseases, Emory University School of Medicine, Atlanta, Georgia, USA; Department of Medicine, Division of Infectious Diseases, Emory University School of Medicine, Atlanta, Georgia, USA; Department of Pathology and Laboratory Medicine, Emory University School of Medicine, Atlanta, Georgia, USA; Department of Medicine, Division of Infectious Diseases, Emory University School of Medicine, Atlanta, Georgia, USA; Georgia Emerging Infections Program, Decatur, Georgia, USA

**Keywords:** clostridioides difficile, fecal microbiota transplantation, recurrent clostridioides difficile, real-world therapy utilization, emerging infections program

## Abstract

**Background:**

Fecal microbiota transplantation (FMT) is recommended for the treatment of recurrent *Clostridioides difficile* infection (rCDI). In the current study, we evaluated rates of rCDI and subsequent FMT in a large metropolitan area. We compared demographic and clinical differences in FMT recipients and nonrecipients and quantified differences in outcomes based on treatment modality.

**Methods:**

A retrospective community-wide cohort study was conducted using surveillance data from the Georgia Emerging Infections Program, the Georgia Discharge Data System, and locally maintained lists of FMTs completed across multiple institutions to evaluate all episodes of *C. difficile* infection (CDI) in this region between 2016 and 2019. Cases were limited to patients with rCDI and ≥1 documented hospitalization. A propensity-matched cohort was created to compare rates of recurrence and mortality among matched patients based on FMT receipt.

**Results:**

A total of 3038 (22%) of 13 852 patients with CDI had rCDI during this period. In a propensity-matched cohort, patients who received an FMT had lower rates of rCDI (odds ratio, 0.6 [95% confidence interval, .38–.96) and a lower mortality rate (0.26 [.08–.82]). Of patients with rCDI, only 6% had received FMT. Recipients were more likely to be young, white, and female and less likely to have renal disease, diabetes, or liver disease, though these chronic illnesses were associated with higher rates of rCDI.

**Conclusions:**

These data suggest FMT has been underused in a population-based assessment and that FMT substantially reduced risk of recurrence and death.

*Clostridioides difficile* infection (CDI) is one of the most common healthcare-associated infections worldwide. The pathogenesis of CDI is linked to metabolism of bile acids performed by microbial communities that reside in the gut (ie, microbiota). Disruption in the typical structure and metabolic functions performed by the microbiota is referred to as *dysbiosis* [1, 2]. Dysbiosis can be longstanding, often resulting in serial CDI infections separated by weeks or months [3]. Professional society guidelines recommend antibiotic treatment with fidaxomicin, or alternatively vancomycin, for the first occurrence of CDI [4]. However, antibiotic treatment is frequently followed by recurrent infection since antibiotics may eliminate commensal bacteria as well as the *C. difficile,* which perpetuates gut dysbiosis. Due to these described mechanisms, approximately 20% of patients with a single episode of CDI will go on to have another CDI episode, also known as recurrent CDI (rCDI) [5]. The likelihood of recurrence increases with each sequential episode of CDI [6].

Treatment of rCDI with live biotherapeutic products can prevent recurrence after antibiotic treatment. These typically comprise live bacteria or bacterial spores derived from healthy, noninfected patients in order to replenish colonic commensal bacteria [7]. Novel live biotherapeutic products have been recently approved by the Food and Drug Administration; during our study period, however, the only one endorsed by society guidelines was fecal microbiota transplantation (FMT). In FMT, stool from a healthy donor is introduced to the patient's colon, usually through colonoscopy, allowing for repopulation of the affected colon with healthy microbiota [4]. Reported efficacy rates of approximately 90% for prevention of rCDI have motivated professional society recommendations to consider FMT for patients with ≥2 recurrences, following completion of a course of standard-of-care antibiotics [4, 8]. In randomized, controlled studies, patients treated with FMT have lower rates of recurrent infection than those treated with antibiotics alone (pooled relative risk, 0.38 [95% confidence interval (CI), 16–.87]) [9]. Despite these benefits, few studies quantify how this therapy is used on a community scale.

The current study describes the real-world use of FMT by characterizing the frequency of administration of FMT and the patient population treated. This work also quantifies the beneficial outcomes of FMT, as defined by recurrence and mortality rates attributable to FMT receipt among patients in a large diverse metropolitan area.

## METHODS

### Study Population

The Georgia Emerging Infections Program (EIP) (funded by the Centers for Disease Control and Prevention) conducts active population-based CDI surveillance in the 8-county metro Atlanta area, health district 3 (population 4.2 million in 2019). The Georgia EIP surveillance activities were approved by the Emory University Institutional Review Board (IRB), with waiver of consent and HIPAA authorization in place. The data collection was approved by the Emory University IRB, the Atlanta VA Research and Development Office, and the Grady Memorial Hospital Research Oversight Committee and exempted for review by the Georgia Department of Public Health IRB. This study evaluated adult residents of health district 3 with diagnosed CDI between 1 January 2016 and 31 December 2019. This end date was chosen due to decreased use of FMT during the coronavirus disease 2019 (COVID-19) pandemic.

### Case Definition and Study Cohort

Episodes of CDI were defined as a positive *C. difficile* toxin or molecular assay result in a stool specimen obtained from a person without a prior positive test in the previous 14 days. rCDI was defined as 2 episodes of CDI within 365 days, and multiple rCDI as >2 episodes within 365 days. This prolonged duration was used to capture outcomes from FMT, which was often completed several months after CDI episodes. For this study, case patients were limited to patients with rCDI and ≥1 documented hospitalization in the state of Georgia during 2016–2019 for whom more complete data were available on medical comorbid conditions. Hospitalization ascertainment was performed by linking rCDI cases to the Georgia Discharge Data System (GDDS) (Georgia Hospital Association, Atlanta). This database contains patient demographics, dates of admission and discharge, and up to 10 associated *International Classification of Diseases, Tenth Revision* (*ICD-10*) codes for all overnight hospitalizations in all general acute care, long-term acute care, and inpatient rehabilitation hospitals. Hospitalization encounters eligible for linkage spanned 2015–2020, to allow for 1 year of lead time before the diagnosis and 1 year of follow-up time around the planned study period of 2016–2019.

### Assigning FMT to Specific CDI Episodes

The Atlanta FMT database was created by combining the list of all FMTs completed at 2 large multicampus healthcare organizations. These 2 organizations were estimated to have provided 90% of FMTs to both inpatients and outpatients in the Atlanta Metropolitan area during this study period, based on expert knowledge (by C.K., N.G.) and analysis of FMT specimen distribution during 2016–2018 by the Microbiome Health Research Institute, “OpenBiome,” (Cambridge, Massachusetts). FMT recipients were defined as study patients who received ≥1 FMT between 2016 and 2019. If a patient underwent FMT twice in 8 weeks, the date attributed to the FMT was the later date, and the FMTs were considered a single treatment with multiple doses [10]. CDI episodes immediately preceding an FMT receipt date were considered treated with FMT; all others were presumed treated with antimicrobials alone. Many of the FMT recipients were not residents of the surveillance catchment area: 247 of the 499 (49%) recipients outside the Georgia EIP catchment and were excluded from study. The majority of the remaining 253 patients (150 [59%]), having received an FMT after the second CDI episode, were successfully linked to GDDS and included in the study. Medical records were reviewed of all patients with CDI who received an FMT before episode 3 to the determine rationale for earlier FMT receipt.

### Derivation of Covariates

**Figure 1.**
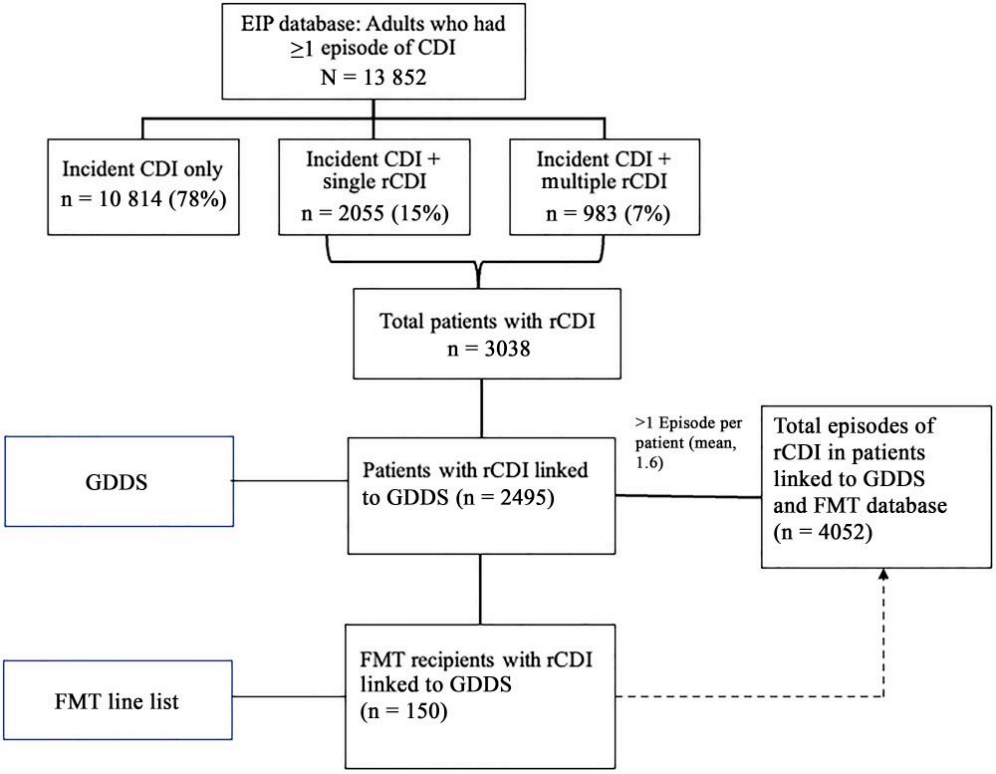
Flow diagram of Emerging Infections Program *Clostridioides difficile* infection (CDI) cases and linkage to external data sets. FMT, fecal microbiota transplantation; GDDS, Georgia Discharge Data System; rCDI, recurrent CDI.

Data were analyzed at an episode level, retaining the sequential episode number and relevant episode-specific data including exposures (characteristics of prior episode, timing between episodes, recent hospitalization data) and outcomes. Episode-specific outcomes were classified as having recurrence if an incident CDI episode occurred within 365 days of the episode. Database linkage and transformation from episode-level to patient-level data are detailed in Figure 1.

Demographic data from the EIP database, including race, were abstracted from the medical record, which relies on institution-specific practices at patient-registration. The all-cause 90-day mortality rate was obtained from Georgia death records (Vital Records, Georgia Department of Public Health). Comorbidity information was assigned to each patient by compiling all *ICD-10* codes associated with any hospitalization during this study period and then using the R comorbidity package to derive values for each of 17 chronic comorbid conditions according to the Charlson comorbidity index [11]. Additional variables for inflammatory bowel disease and hemodialysis were created using *ICD-10* codes K50.9, K51.9, K52.3, K52.9, and Z99.2.

Episode-specific hospitalization exposure variables for each CDI episode were derived using the CDI test date and data in the GDDS. Hospital-onset CDI was defined as CDI test date >3 days after the date of admission. Prior admission was defined as ≥1 admission date ≤90 days before the CDI test date. Readmission was defined as ≥1 new admission within 90 days after the CDI test date but before any subsequent episode of CDI. Recent intravenous antibiotic use was derived using a list of *ICD-10* codes that approximated inpatient antibiotic use [12]. If any of these *ICD-10* codes was associated with an admission ≤90 days before the CDI test date, this CDI episode was assigned to have had recent intravenous antibiotics. A similar approach was used to assign a CDI episode as having intravenous antibiotics after CDI.

### Statistical Analysis

The incidence of rCDI per 100 000 cases per year was calculated using census data for health district 3 (National Vital Statistics System). Demographics, comorbid conditions, and episode-specific exposures were compared between episodes with recurrence and those without recurrence, estimating the parameters of association and standard errors according to the generalized estimating equations approach using PROC GENMOD (SAS), as some patients had multiple episodes. Similar methods were used to identify potential predictors of FMT recipients by comparing these data between CDI episodes treated with an FMT to all others.

A propensity-matched cohort of CDI episodes was constructed matching propensity scores calculated based on demographic and comorbid variables significantly associated with FMT receipt. These included sex, age group, race, episode number, diagnosis of cerebrovascular disease, congestive heart failure (CHF), renal disease, and diabetes. Episodes treated with FMT were propensity matched on a 1:1 basis with non-FMT control episodes using PROC PSMATCH in SAS. We conducted exact matching on episode number because the episode number affects the likelihood of both subsequent recurrence and FMT receipt.

Univariate and multivariate logistic regression analysis was conducted on the propensity-matched cohort. The multivariable analysis assessed the association between the outcome with FMT receipt, adjusting for hospitalization following the CDI episode (as a potential confounder for additional intravenous antibiotics or exposure to *C. difficile)* and propensity-matched factors. Backward selection was used to model the impact of FMT on CDI recurrence and 90-day mortality rates in 2 separate models. Covariates were retained if statistically significant at *P* < .05, and the adjusted odds ratio (aOR) was reported. All outcomes were analyzed used PROC LOGISTIC; CDI episodes in the propensity-matched cohort were all from unique patients.

## RESULTS

### Overview of Patients With CDI

**Figure 2.**
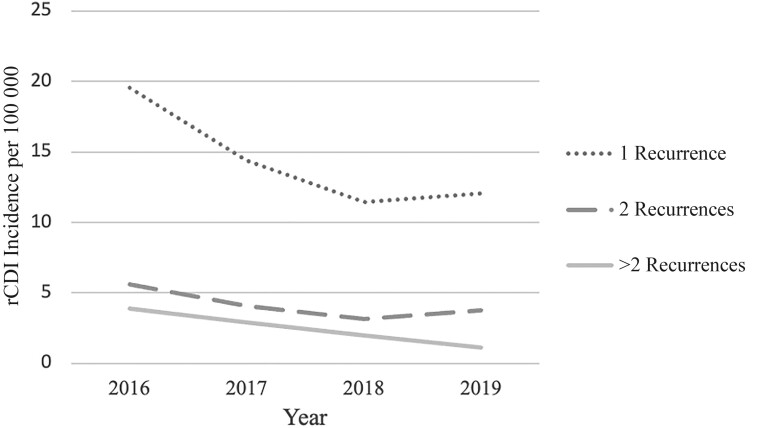
Crude annual incidence of recurrent *Clostridioides difficile* infection (rCDI) per 100 000 population in the Atlanta metropolitan area, 2016–2019.

A total of 13 852 adults were identified as having ≥1 episode of CDI in the greater Atlanta metropolitan area between 2016 and 2019. Of these patients, 10 814 (78%) had 1 CDI episode, 2055 (15%) had 2 episodes in a year, and 983 (7%) had multiple recurrences (Supplementary Table 1). The incidence of rCDI decreased yearly during the study period (Figure 2). Among patients with any rCDI, the mean duration between the first 2 episodes was longer in patients who had 2 episodes than in those with multiple recurrences (82 vs 65 days, respectively; *P* < .01) (Supplementary Table 1).

### Predictors of rCDI

Of patients with ≥1 rCDI episode, 82% were linked to GDDS. The 2495 patients with ≥1 GDDS recorded hospitalization and 1 episode of rCDI during this study period experienced 1557 subsequent CDI episodes for a total of 4052 distinct rCDI episodes for analysis (Table 1). Apart from slight differences in age distribution, demographics were similar between the episodes with a further recurrence and those without (Table 1). However, chronic pulmonary disease, CHF, mild liver disease, diabetes with complications, renal disease, and dialysis were more common among episodes with recurrence (Table 1). Rehospitalization within 90 days of CDI was associated with a significantly higher odds of recurrence (odds ratio [OR], 1.78 [95% CI, 1.36–2.34]) (Table 1).

**Table 1.** Demographic and Clinical Characteristics Associated With *Clostridioides difficile* Infection Episodes by Recurrence Status Within 365 Days

Characteristic	Patients, No. (%)		OR (95% CI)
	No Recurrence Within 365 d (n = 2429)	Recurrence Within 365 d (n = 1623)	
Race			
White	1057 (44)	770 (47)	Reference
Black	872 (36)	606 (37)	0.70 (.49–1.02)
Other	34 (1.4)	21 (1.3)	0.93 (.23–3.73)
Unknown	466 (19)	226 (14)	0.41 (.25–.66)
Sex			
Female	1446 (60)	955 (59)	Reference
Male	983 (40)	668 (41)	0.95 (.82–1.10)
Age category			
18–44	344 (14)	334 (21)	Reference
45–64	794 (33)	524 (32)	0.60 (.36–1.02)
65–79	821 (34)	488 (30)	0.60 (.36–1.01)
≥80	470 (19)	277 (17)	0.52 (.3–.933)
Comorbid conditions			
Peripheral vascular disease	298 (12)	203 (13)	1.15 (.93–1.41)
Cerebrovascular disease	449 (18)	255 (16)	0.97 (.81–1.17)
Dementia	337 (14)	206 (13)	0.97 (.78–1.19)
Chronic pulmonary disease	630 (26)	517 (32)	1.28 (1.09–1.5)
Congestive heart failure	1003 (41)	780 (48)	1.34 (1.16–1.55)
Rheumatoid disease^a^	134 (6)	96 (6)	1.05 (.77–1.44)
Peptic ulcer disease	187 (8)	159 (10)	1.23 (.96–1.60)
Mild liver disease	262 (11)	235 (14)	1.29 (1.03–1.61)
Diabetes without complications	805 (33)	592 (36)	1.12 (.95–1.29)
Diabetes with complications	687 (28)	565 (35)	1.25 (1.06–1.46)
Hemiplegia or paraplegia	185 (8)	104 (6)	0.97 (.75–1.27)
Renal disease	973 (40)	813 (50)	1.45 (1.25–1.67)
Cancer	446 (18)	273 (17)	0.91 (.76–1.10)
Moderate to severe liver disease	98 (4)	68 (4)	1.04 (.73–1.49)
HIV	89 (4)	78 (5)	1.06 (.72–1.56)
Inflammatory bowel disease	14 (0.6)	35 (2.16)	1.37 (.50–3.78)
Dialysis	145 (6)	179 (11)	1.82 (1.40–2.37)
Exposures before (within 90 d) or during episode			
Recent hospital admission	1747 (72)	1114 (69)	0.92 (.63–1.34)
Recent intravenous antibiotics	1108 (46)	648 (40)	0.90 (.64–1.28)
Onset during hospital admission	256 (11)	141 (9)	0.82 (.46–1.49)
Exposures within 90 d after episode			
Readmission	831 (34)	818 (50)	1.78 (1.36–2.34)
Intravenous antibiotics	488 (20)	429 (26)	1.29 (.93–1.77)
FMT received	99 (4)	51 (3)	0.76 (.54–1.08)

Abbreviations: CI, confidence interval; FMT, fecal microbiota transplantation; HIV, human immunodeficiency virus; OR, odds ratio.

^a^Rheumatoid arthritis/collagen vascular disease.

### Timing and Indication for FMT

A total of 293 FMTs were administered to 250 individual residents of health district 3. The first FMT was administered at different time points, with 127 (52%) administered before episode 3 (52 [22%] before and 75 [30%] at episode 2), 68 (27%) at episode 3, and 51 (20%) after subsequent episodes. Among all 13 852 patients, FMT receipt rates were therefore 0.4% (52 of 13 852) before 2, 2.5% (75 of 3038) at episode 2, 7% (68 of 983) at episode 3, and 7% (51 of 729) among later episodes. Although the most common indication (54%) for FMT was multiple rCDI, 13 patients (5%) received FMT after symptom resolution but before episode 3, 42 (16%) were treated for refractory symptoms (antimicrobial treatment failure), and for 49 (19%) were treated for an indication that remained uncertain after record review (Supplementary Table 2).

### Predictors of Episodes Receiving FMT

Among FMT recipients, 150 had ≥1 overnight hospitalization and were included in the episode-level analysis and final study cohort. Patients who received FMT were more likely to be white, female, and <64 years of age (Table 2). Patients with chronic comorbid conditions—specifically cerebrovascular disease, CHF, diabetes with complications, and renal disease—were less likely to have episodes treated with FMT than those without those conditions (Table 2). The odds of FMT treatment were higher as the serial number of recurrences increased; the OR was 13.9 (95% CI, 5.7–34.1) for third and 26.7 for fourth episodes (9.1–79.0) (Table 2).

**Table 2.** Predictors of Receipt of Fecal Microbiota Transplantation in *Clostridioides difficile* Infection Episodes Among Patients With ≥1 Recurrence and Hospitalization

Predictor	Patients by Receipt of FMT After CDI Episode, No. (%)		OR (95% CI)
	No FMT (n = 3902)	FMT Received (n = 150)	
Demographic characteristics			
Race			
White	1737 (45)	90 (60)	Reference
Black	1444 (37)	34 (23)	0.19 (.08–.46)
Other	52 (1.3)	3 (2)	1.25 (.09–18.60)
Unknown	669 (17)	23 (15)	0.42 (.15–1.19)
Sex			
Female	2292 (59)	109 (72)	Reference
Male	1610 (41)	41 (27)	0.55 (.39–.78)
Age category			
18–44 y	641 (16)	37 (25)	Reference
45–64 y	1258 (33)	50 (33)	0.40 (.16–1.02)
65–79 y	1272 (33)	37 (25)	0.22 (.08–.60)
≥80 y	721 (18)	26 (17)	0.35 (.11–1.07)
Comorbid conditions			
Peripheral vascular disease	488 (13)	13 (9)	0.67 (.38–1.17)
Cerebrovascular disease	703 (18)	11 (7)	0.35 (.19–.65)
Dementia	531 (14)	12 (8)	0.56 (.31–1.00)
Chronic pulmonary disease	1106 (28)	41 (27)	0.98 (.69–1.39)
Congestive heart failure	1739 (45)	44 (29)	0.55 (.39–.77)
Rheumatoid disease	220 (6)	10 (7)	1.24 (.68–2.26)
Peptic ulcer disease	336 (8.6)	10 (7)	0.77 (.41–1.44)
Mild liver disease	487 (12)	10 (7)	0.51 (.27–.97)
Diabetes	1363 (35)	34 (23)	0.54 (.37–.79)
Diabetes with complications	1229 (32)	23 (15)	0.41 (.27–.64)
Hemiplegia or paraplegia	283 (7)	6 (4)	0.53 (.23–1.19)
Renal disease	1749 (45)	37 (25)	0.42 (.29–.6)
Cancer	698 (18)	21 (14)	0.78 (.50–1.22)
Moderate to severe liver disease	165 (4.2)	1 (0.7)	0.14 (.02–1.0)
HIV	163 (4)	4 (3)	0.75 (.31–1.78)
Inflammatory bowel disease	48 (1)	1 (0.01)	0.52 (.03–2.5)
Dialysis	318 (4)	6 (4)	0.47 (.18–.98)
CDI episode			
2	2442 (63)	53 (35)	Reference
3	806 (21)	55 (37)	13.9 (5.66–34.14)
4	326 (8)	30 (20)	26.7 (9.06–78.81)
5	156 (4)	5 (3)	2.25 (.24–20.65)
>5	172 (4)	7 (5)	3.32 (.50–22.00)

Abbreviations: CDI, *Clostridioides difficile* infection; CI, confidence interval; FMT, fecal microbiota transplantation; HIV, human immunodeficiency virus; OR, odds ratio.

### Estimating Outcomes Attributed to FMT

**Figure 3.**
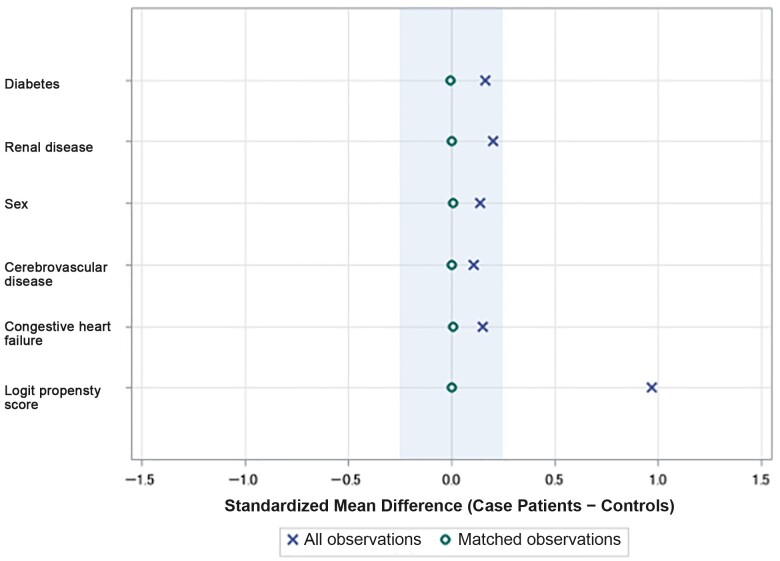
Standardized mean differences between case patients (who received fecal microbiota transplantation) and controls (who did not) in the propensity-matched cohort, compared with all observations. Larger deviations for zero difference indicate difference in frequency of characteristics between case patients and controls. A difference of zero for matched observations indicates no difference in the frequency of each characteristic between case patients and matched controls.

In unadjusted analysis, the association of FMT receipt with decreased CDI recurrence did not reach statistical significance (OR, 0.76 [95% CI, .54–1.08]) (Table 1). Propensity matching was performed with good matching between 150 case episodes and 150 control episodes (Figure 3 and Supplementary Table 3). In the propensity-matched analysis, FMT receipt was protective against recurrence. Among episodes treated with FMT, 51 (34%) had recurrence, compared with 69 (46%) of matched controls without FMT (OR, 0.60 [95% CI, .38–.96]). A multivariate analysis adjusted for readmission after CDI and FMT receipt, and FMT receipt remained significantly associated with lower odds of subsequent recurrence (aOR, 0.6 [95% CI, .38–.97]) (Table 3).

**Table 3.** Outcomes by Fecal Microbiota Transplantation Receipt in Propensity-Matched Cohort: Multivariate Analysis

Outcome	Patients, No. (%)		OR (95% CI)	aOR (95% CI)^a^
	FMT Not Received (n = 150)	FMT Received (n = 150)		
rCDI	69 (46)	51 (34)	0.60 (.38–.96)	0.61 (.38–.97)
90-d mortality rate	14 (9)	4 (3)	0.26 (.09–.83)	0.26 (.08–.82)

Abbreviations: aOR, adjusted odds ratio; CI, confidence interval; FMT, fecal microbiota transplantation; OR, odds ratio; rCDI, recurrent *Clostridioides difficile* infection.

^a^The covariates in the multivariate model for rCDI were: readmission within 90 days, FMT receipt, and propensity score and for 90-day mortality were: congestive heart failure, FMT receipt, and propensity score.

Eighteen of 300 patients (6%) in the propensity-matched cohort died within 90 days of the CDI episode date. The all-cause 90-day mortality rate was higher in propensity-matched patients who did not receive FMT than in those who did (14 of 150 patients [9%] vs 4 of 150 [3%], respectively). In a multivariate model of the association of FMT exposure and 90-day mortality, CHF was the only underlying comorbid condition that was significant by backward selection. In this model, FMT receipt was significantly associated with a lower odds of death within 90 days, with an aOR of 0.26 (95% CI, .08–.82) (Table 3).

## DISCUSSION

This study describes the incidence of rCDI and the use of FMT in a large metropolitan area across multiple healthcare organizations and quantifies the risk-adjusted benefit of FMT. Among the propensity-matched cohort, FMT receipt was found to be protective against recurrence (aOR, 0.6). The rate of CDI resolution at 1 year after FMT was 66%. The majority of the FMT recipients in this cohort (87%) received a single infusion of FMT; 4% of patients received 2 FMTs within 8 weeks, which was considered a single treatment. Previous reports suggest that patients with rCDI receiving a single infusion have rates of resolution from 49% to 95% [13]. This heterogeneity in resolution likely reflects both different definitions of resolution (eg, in our study, this was defined as no further positive test results within 365 days, as we were unable to determine symptomatic cure), variability in study design and quality, as well as the heterogeneity of the product itself, given each dose is derived from different donors with different microbiota.

In addition to lower rates of recurrence, patients who received FMT had a lower all-cause 90-day mortality rate than propensity-matched patients without FMT receipt. This finding is consistent with other studies that have shown lower all-cause mortality rates after FMT in both patients with rCDI and those with severe infection [14, 15]. This may be related to fewer complications associated with CDI, such as bowel perforation and bloodstream infections, as well as some practitioner reluctance to perform this procedure in patients with a high mortality risk [14]. This study builds on prior work through the use of propensity score matching, surveillance data from an 8-county metropolitan area, and treatment data from multiple medical centers. It also provides insight on real-world use of FMT on a community scale.

In spite of the observed lower rates of recurrence and recommendations for the use of FMT after episode 3 in national professional society guidelines on rCDI treatment, a very small proportion of patients with rCDI received an FMT. Of all patients with rCDI only 6% received a recorded FMT in this study. Among patients with ≥3 episodes of CDI, the point at which guidelines recommend consideration of FMT, 12% received an FMT after episode 3. The low rate of administration likely reflects a combination of several barriers to FMT administration, including provider familiarity with the procedure and understanding of where to refer patients to receive FMT, difficulty of insurance coverage, concerns about subjecting patients to an invasive procedure, and, possibly, patient perceptions of the procedure. While many of the studies evaluating provider and patient attitudes toward FMT predate our study, in a systematic review providers cited some concerns about infection transmission in more chronically ill patients, concern about patient acceptability, and difficulty accessing this therapy as a reason they would not refer patients to receive an FMT [16, 17].

FMT receipt was disproportionately administered to some groups compared with others. Female patients were more likely to have an incident case of CDI but not appreciably more likely to have a recurrent episode. This sex difference in incident CDI has been reported in multiple studies, though the mechanism has yet to be elucidated [18, 19]. While rates of recurrence do not differ appreciably between female and to male patients, female patients were more likely to have received FMT in this study. FMT was administered more often to younger patients and patients without several serious comorbid conditions, such as cerebrovascular disease, CHF failure, renal disease, diabetes, and liver disease. Given that all FMTs in this study were administered through colonoscopy, physicians may have been reluctant to consider this therapy in older patients or those with comorbid conditions, as FMT may have been considered a higher-risk procedure compared with antibiotic administration. In terms of race, while white and black patients experienced rCDI at similar rates, white patients were more likely than black patients to have received FMT, demonstrating racial disparities in FMT receipt. Notably, FMT was not offered at the large safety net hospital in Atlanta during this study period and was offered only at private and academic centers.

Several comorbid conditions were found to be risk factors for CDI recurrence, including chronic pulmonary disease, CHF, liver disease, diabetes with complications, renal disease, and use of hemodialysis. These conditions commonly require hospitalization or frequent visits to healthcare facilities, which could increase risk of exposure to CDI and possibly antibiotics as well. While cancer and inflammatory bowel disease are both often cited as risk factors for CDI or rCDI, this was not confirmed in our study. In the case of inflammatory bowel disease, there were few patients with this condition represented in our cohort. Finally, gastric acid suppression is often cited as a risk factor for rCDI [6, 20, 21]. This could not be directly measured using these data sets; however, peptic ulcer disease, a condition for which gastric acid suppression is a mainstay of therapy, also was not associated with increased risk of rCDI. Admission following CDI was found to be a significant risk factor for recurrence. However, our proxy metric for inpatient intravenous antibiotic use in the 90 days following CDI as well as hospital-onset CDI were not predictive of recurrence, though these are often cited as risk factors for CDI [20, 21].

One of the most important findings in this study is that older patients (aged >65 years) and patients with liver disease, diabetes, or renal disease were both more likely to experience rCDI and less likely to receive FMT, the therapy that has a sustained cure rate for CDI superior to that of antibiotics alone. We hypothesize that the lower rates of FMT in these groups are due to the invasive nature of this therapy. While FMT has generally been shown to be safe in patients with significant comorbid conditions, such as organ transplantation and cirrhosis [22, 23], there have been reports of infection transmission through FMT [24]. Therapeutics approved by the Food and Drug Administration include an oral product that uses bacterial spores derived from donor stool and an enema with a known consortia of donor microbiota [25–28]. Both of these products may be a preferred option for patients with multiple comorbid conditions, as they do not require anesthesia or invasive procedures. Further work is needed to evaluate access to these commercial alternatives to FMT and whether they are associated with similar real-world reductions in recurrence and 90-day mortality rates.

This work expands our understanding of real-world use of FMT, including variations in practices from guidelines and demographic and clinical differences in FMT-eligible patients who were or were not offered FMT. Given that many studies evaluating FMT outcomes are restricted to single institutions, this study allowed for capturing of data on a community-level basis, enabling the evaluation of practice patterns across multiple institutions. This study identified a 22% recurrence rate among all patients with CDI, similar to that in other studies, suggesting that these data may be generalizable for the United States [29].

The current study had several limitations. Given that the study used surveillance data and could not capture information about diarrheal symptoms, it is likely that some positive test results included in the analysis did not correspond with clinical disease; this is a limitation of all CDI studies completed through the Emerging Infections Program. We used a longer interval between episodes (within 365 days) than surveillance definitions, which usually cite 8 weeks between episodes to qualify rCDI. This was done because the time between episodes in patients who were offered FMT much exceeded 8 weeks in many cases, and we believe that this metric more accurately enables characterization of the population who received FMT in our real-world setting. Other real-world studies also evaluate longer durations between episodes [30, 31]. However, owing to this departure from the surveillance definition, the recurrence rate cited in our study may overestimate rates based on more standard metrics.

Since one of the Atlanta-area institutions did not keep a systematic record of patients having received FMT, there was some risk of misclassification bias; some patients classified as nonrecipients may have had an FMT. However, the total number of FMTs performed by this institution in patients who could have been classified as nonrecipients would have constituted <10% of FMTs provided in this time period. There were 3902 candidate control episodes, 150 (4%) of which were used in the final propensity score analysis, so the chances of misclassification in the propensity-matched model is relatively low. A small proportion of patients (4%) had serial FMTs within an 8-week window, which we analyzed as a single treatment date, with some potential bias toward more efficacy of an FMT treatment. In addition, information about comorbid conditions was restricted to *ICD-10* codes entered by inpatient physicians, which may not include all of a patent’s comorbid conditions. Similarly, prior or subsequent intravenous antibiotic use was approximated from mapping encounter-specific *ICD-10* codes, which may have allowed for some misclassification of patients who did or did not actually receive antibiotics, though our numbers are in keeping with prior data that 50%–55% of patients will receive ≥1 intravenous antibiotic during hospitalization [32, 33]. Finally, we were unable to capture outpatient use of antibiotics in this study, which is a risk factor for CDI.

Despite these limitations, the current study provides an estimate of the benefits in terms of reduction in the risk of recurrence (approximately 40% lower odds) and the mortality rate among patients receiving microbiota replacement therapy outside clinical studies before the COVID-19 pandemic and before treatment guidelines placed more importance on these therapies. The low frequency of FMT administration among FMT-eligible patients with rCDI highlights a need for further attention to access to microbiota therapies.

## Supplementary Material

ofae309_Supplementary_Data

## Supplementary Data

Supplementary materials are available at *Open Forum Infectious Diseases* online. Consisting of data provided by the authors to benefit the reader, the posted materials are not copyedited and are the sole responsibility of the authors, so questions or comments should be addressed to the corresponding author.
