# Supplementary material for: Recurrent Clostridioides difficile Infection and Outcome of Fecal Microbiota Transplantation Use: A Population-Based Assessment
Source: Open Forum Infect Dis. 2024 Jun 15;11(7):ofae309. doi: 10.1093/ofid/ofae309 (PMC11227225; doi:10.1093/ofid/ofae309)
Supplement: ofae309_Supplementary_Data [file ofae309_supplementary_data.docx]

**Supplemental Tables**

**Table S1.** Demographic and Baseline Characteristics of 13,852 adults having at least one CDI episode in the Atlanta Metropolitan area (regardless of hospitalization in this period), by recurrence category, 2016-2019

|  | No Recurrence | Single recurrence | Two or more recurrence | p-value* |
| --- | --- | --- | --- | --- |
|  | N=10,814 | N=2,055 | N=983 |  |
| Patient Characteristic | N (%) | N (%) | N (%) |  |
| Sex |  |  |  |  |
| Female | 6436 (60%) | 1233 (60%) | 604 (61%) | 0.28 |
| Male | 4378 (40%) | 822 (40%) | 379 (39%) |  |
| Age Category (years) |  |  |  |  |
| 18-44 | 2338 (21%) | 329 (16%) | 163 (17%) | <0.01 |
| 45-64 | 3546 (32%) | 665 (32%) | 314 (32%) |  |
| 65-79 | 3202 (30%) | 682 (33%) | 332 (33%) |  |
| 80+ | 1728 (16%) | 379 (18%) | 174 (18%) |  |
| Race |  |  |  | <0.01 |
| White | 4236 (39%) | 825 (40%) | 464 (47%) |  |
| Black | 3445 (32%) | 705(34%) | 324 (33%) |  |
| Other | 238 (2%) | 41 (2%) | 14(1%) |  |
| Days between Episode 1 and 2, mean (SD) | N/A | 89 (114) | 65 (59) | <0.001** |

*Chi square test across all groups; ** ANOVA

**Table S2:** Characteristics of FMT receipt among all residents in Health District 3 (Atlanta Metropolitan area) between 2016-2019

|  | Healthcare System A | Healthcare System B | Total |
| --- | --- | --- | --- |
|  | N (%) | N (%) |  |
| Total FMTs | 174 | 119 | 293 |
| Individuals | 144 | 106 | 250 |
| Indications for the first FMT by individual |  |  |  |
| At or after episode 3 | 65 (43%) | 69 (65%) | 134 (52%) |
| At least one episode found outside of catchment | 6 (4%) | 6 (6%) | 12 (5%) |
| FMT administered after resolution of symptoms, but before episode 3 | 4 (3%) | 9 (8%) | 13 (5%) |
| Refractory^ | 30 (20%) | 12 (11%) | 42 (16%) |
| Unknown^&^ | 39 (26%) | 10 (9%) | 49 (19%) |
| Timing of multiple FMTs |  |  |  |
| All FMTs before episode 3 | 3 (13%) | 1 (8%) | 4 (11%) |
| All FMTs after episode 3 | 13 (54%) | 9 (75%) | 22 (61%) |
| Combination of FMTs before and after episode 3 | 8 (33%) | 2 (17%) | 10 (28%) |

^ Refractory: continued diarrhea despite completion of course of antibiotics

^&^ unknown: unable to find exact indication on chart review

## **Table S3:** Baseline Characteristics in Propensity Score-Matched Controls and Cases, matched on likelihood for CDI episodes receiving FMT: Propensity Matched Cohort

|  | No FMT Received after Episode | FMT Received after Episode |  |
| --- | --- | --- | --- |
| Characteristic | **N = 150** | **N = 150** | P-value |
| Episode Number |  |  | >0.9 |
| 2 | 53 | 53 |  |
| 3 | 55 | 55 |  |
| 4 | 30 | 30 |  |
| 5 | 5 | 5 |  |
| >5 | 7 | 7 |  |
| Gender |  |  | >0.9 |
| Female | 109 | 109 |  |
| Age Category |  |  | >0.9 |
| 18-44 | 38 | 37 |  |
| 45-64 | 49 | 50 |  |
| 65-79 | 36 | 37 |  |
| 80+ | 27 | 26 |  |
| Race |  |  | >0.9 |
| White | 93 | 90 |  |
| Black | 33 | 34 |  |
| Other | 1 | 3 |  |
| Comorbid Conditions |  |  | >0.9 |
| Cerebrovascular Disease | 11 | 11 |  |
| CHF | 45 | 44 |  |
| Renal Disease | 37 | 37 |  |
| Diabetes with complications | 22 | 23 |  |
